# Submillisecond *in situ* X-ray diffraction measurement system with changing temperature and pressure using diamond anvil cells at BL10XU/SPring-8

**DOI:** 10.1107/S1600577523010974

**Published:** 2024-02-19

**Authors:** Saori Kawaguchi-Imada, Ryosuke Sinmyo, Kenji Ohta, Shogo Kawaguchi, Toshiyuki Kobayashi

**Affiliations:** a Japan Synchrotron Radiation Research Institute, 1-1-1 Kouto, Sayo-cho, Sayo-gun, Hyogo 679-5198, Japan; b Department of Physics, School of Science and Technology, Meiji University, 1-1-1 Higashi Mita, Tama-ku, Kawasaki, Kanagawa 214-8571, Japan; c Department of Earth and Planetary Sciences, Tokyo Institute of Technology, 2-12-1 Ookayama, Meguro, Tokyo 152-8551, Japan; Advanced Photon Source, USA

**Keywords:** diamond anvil cells, X-ray diffraction, *in situ* time-resolved measurement, beamlines

## Abstract

Recently, there has been a high demand for elucidating kinetics and visualizing reaction processes under extreme dynamic conditions, such as chemical reactions under meteorite impact conditions, structural changes under non­equilibrium conditions, and *in situ* observations of dynamic changes. To accelerate material science studies and Earth science fields under dynamic conditions, a submillisecond *in situ* X-ray diffraction measurement system has been developed using a diamond anvil cell to observe reaction processes under rapidly changing pressure and temperature conditions replicating extreme dynamic conditions. The development and measurements were performed at the high-pressure beamline BL10XU/SPring-8 by synchronizing a high-speed hybrid pixel array detector, laser heating and temperature measurement system, and gas-pressure control system that enables remote and rapid pressure changes using the diamond anvil cell. The synchronized system enabled momentary heating and rapid cooling experiments up to 5000 K via laser heating as well as the visualization of structural changes in high-pressure samples under extreme dynamic conditions during high-speed pressure changes.

## Abstract 2

A submillisecond X-ray diffraction measurement system targeted at microscopic samples in a diamond anvil cell has been developed at BL10XU/SPring-8. This system has enabled the visualization of structural changes of high-pressure samples in the diamond anvil cell during instantaneous heating and quenching experiments combined with laser heating and during instantaneous compression and decompression experiments using a two-line gas-pressure control system, with a resolution in the submillisecond range.

## 1. Introduction

By utilizing the high-brilliance and high-energy X-rays from SPring-8, the users of BL10XU/SPring-8 have published various research results, spanning the fields of basic science (Sakai *et al.*, 2020 ▸; Akahama *et al.*, 2021 ▸) and deep Earth science (Kuwayama *et al.*, 2020 ▸; Tagawa *et al.*, 2021 ▸) to material science, including the search for room-temperature superconductors (Einaga *et al.*, 2016 ▸; Song *et al.*, 2023 ▸). However, while numerous experimental studies have been conducted under static pressure conditions, the development of a dynamic measurement system targeting high-pressure samples in diamond anvil cells (DACs) has just begun. Dynamic high-pressure events are fundamental processes for understanding the evolution of Earth and other planets. The origin of the primitive Earth can be attributed to repeated collisions with smaller celestial bodies (Wood *et al.*, 2006 ▸). After growing to a certain size, the Earth is believed to have been almost or completely molten owing to the impact of a huge Mars-sized celestial body (Canup & Asphaug, 2001 ▸). Later, it was also hypothesized that numerous meteorites impacted Earth during the Late Veneer, introducing volatile elements (Brasser *et al.*, 2016 ▸). A further prevailing theory is that the collisions of meteorites and small bodies that occurred during that period are considered to be the origin of water (Sakuraba *et al.*, 2021 ▸) and amino acids (Furukawa *et al.*, 2009 ▸; Callahan *et al.*, 2011 ▸; Martins *et al.*, 2013 ▸) on Earth. The experimental reproduction of dynamic environments, such as those observed in the case of meteorite impacts, is crucial to understand how the Earth melted, solidified and grew, and to understand the origin of water and life.

Currently, the combination of shock compression experiments and X-ray free-electron laser (XFEL) is the mainstream method for rapidly evaluating the behavior of materials under dynamic high-temperature and pressure conditions (Okuchi *et al.*, 2021 ▸). Meanwhile, the *in situ* observation of complex reactions and phenomena after small celestial body collisions, along with the alterations in temperature and pressure, is equally important along with the pump–probe measurements using XFELs for gaining a deeper understanding of material behavior. Based on the analysis of Yamato 791384, a meteorite impact with a ∼10 km diameter indicates that the instantaneous temperature and pressure values at the location of impact are ∼2500 K and 20 GPa, respectively (Ohtani *et al.*, 2004 ▸). Furthermore, the diffusion of metal ions in the Tenham meteorite indicates that a high-temperature and pressure state persisted for milliseconds to seconds after the impact (Beck *et al.*, 2005 ▸). Previous studies on meteorites revealed that shocked meteorites contained enigmatic high-pressure minerals, such as seifertite and lonsdaleite (El Goresy *et al.*, 2008 ▸; Bundy & Kasper, 1967 ▸). The estimated peak pressure of the impact was ∼25 GPa (El Goresy *et al.*, 2008 ▸); however, seifertite (SiO_2_ in α-PbO_2_-type crystal structure) is formed at a considerably higher pressure (>100 GPa) in static high-pressure experiments (Murakami *et al.*, 2003 ▸). This discrepancy may be attributed to the partial rearrangements of atoms during phase transition due to the kinetics (Kubo *et al.*, 2015 ▸; Černok *et al.*, 2017 ▸) but remains unclear thus far. Even during giant impacts the temperature is extremely high, instantaneously exceeding 5000 K; however, the collision bodies and the Earth will cool down subsequently to an equilibrium state over time, experiencing various temperature and pressure histories (Hosono *et al.*, 2019 ▸). After the collision, small celestial bodies can sustain reaction times of at least submilliseconds, leading to complex reactions. According to the smoothed particle hydro­dynamics calculation simulating the impact of comets on a sea, the impactor comets exhibited various pressure–temperature passes between 0–160 GPa and 2000–12000 K within 1 s because of multiple collisions between ejected particles (Pierazzo & Chyba, 1999 ▸). It is not straightforward to reproduce such pressure–temperature passes by conventional shock experiments. Furthermore, experiments using DACs are suited to reproduce events with relatively mild temperatures and pressures and reactions with durations of milliseconds or longer.

In materials research, in the search for new highly performing materials, there is growing interest in establishing synthetic processes that do not rely on serendipity. To achieve this goal, there is a high demand for elucidating the generation mechanism of synthesized material and visualizing kinetics and reaction processes through temperature–pressure quenching. High-speed X-ray diffraction (XRD) measurements during rapid pressure changes are being performed at various synchrotron radiation facilities worldwide, such as the Advanced Photon Source (Tomasino & Yoo, 2017 ▸) and PETRA III at Deutsches Elektronen-Synchrotron (DESY) (Jenei *et al.*, 2019 ▸, Otzen *et al.*, 2023 ▸). Additionally, some studies involving time-resolved XRD measurements during laser heating are in progress (Yoo *et al.*, 2012 ▸; Konôpková *et al.*, 2021 ▸). However, a measurement system that can perform *in situ* time-resolved XRD measurements on a submillisecond scale under changing pressure and temperature has not been developed to date.

Therefore, we initiated the development of submillisecond *in situ* XRD measurement techniques with varying pressure and temperature conditions using DACs at BL10XU/SPring-8 with the aim of visualizing rapid structural changes under extreme dynamic conditions, such as those encountered during meteorite impacts. Herein, we present our results pertaining to the development of a submillisecond XRD measurement system with changing temperature and pressure using DACs and the experimental results obtained from it. All components of this system, including the high-speed hybrid pixel array X-ray detector LAMBDA 750k, laser heating and temperature measurement system optimized for stable heating and high-speed temperature measurement, and newly developed gas-pressure control system for high-speed pressure changes, can be controlled in a synchronized manner using an external trigger. Our development enables *in situ* observation of structural changes via XRD measurement during temperature and pressure changes through instantaneous laser heating and quick compression–decompression experiments.

## 2. Experimental development and methods

### 2.1. Development of the high-speed XRD measurement system combined with microfocus X-rays

To perform time-resolved XRD measurements on small DAC samples, three key technologies/instruments are required: (i) a high-speed detector for signal detection, (ii) high-flux focused X-rays, and (iii) a well developed sample environment to realize dynamic and extreme conditions. The installation of these hardware components is not the only critical task, and software development is also of substantial importance. This is because without synchronizing all the hardware through software, the system cannot be properly established. To enable synchronization between the instruments, a new control system based on LabVIEW and Python was developed to unify the instrument control system. By unifying the control systems that were previously scattered and mixed, all instruments could be operated from a single control computer, resulting in increased efficiency and convenience during beam-time usage.

A high-speed X-ray detector must be installed. An imaging plate (R-AXIS IV++, Rigaku) and flat-panel detectors (XRD0822, Perkin Elmer) were introduced in the experimental hutch 2 of BL10XU/SPring-8. While the imaging plate detector had a readout resolution of 100 µm, large area of 300 mm × 300 mm and wide dynamic range of 20 bits, it required at least 3 min for exposure and recording reading. Alternatively, the flat-panel detector was highly sensitive to high-energy X-rays and had a maximum frame rate of 25 Hz. However, this rate is insufficient for millisecond measurements and the acquisition of small signals at high speed is further complicated by dark current noise during readout. Therefore, it is impossible to perform high-speed measurements at submillisecond levels with conventional detectors.

Recently, the high-speed hybrid pixel array detector LAMBDA 750k was introduced at BL10XU/SPring-8, which is based on Medipix3 technology. The LAMBDA 750k (X-Spectrum GmbH) detector is a commercialized version of the previously reported one (Pennicard *et al.*, 2014 ▸). We selected a CdTe-type sensor with high quantum efficiency for high-energy X-rays with energies above 30 keV. The pixel size is 55 µm × 55 µm and provides high angular resolution. The main advantages of the LAMBDA 750k detector are its noise-free photon counting capability and high readout speed. In this study, we used continuous read–write mode with 12-bit counter depth to record data up to 2000 frames s^−1^. The maximum pixel count is below 200 counts in millisecond high-speed measurements conducted in 12-bit mode. Consequently, afterimages caused due to saturation have no effect on any data presented in this study. The LAMBDA 750k detector is controlled by a Python script and LabVIEW software.

The introduction of the LAMBDA 750k detector completes the preparation for high-speed XRD measurements. Subsequently, we present the specifications of the X-rays used at BL10XU/SPring-8. High-brilliance and high-energy synchrotron X-rays are supplied from an insertion device. In the experiments, the undulator X-rays were monochromated to 30 keV using liquid-nitro­gen–cooled Si (111) double crystals. The energy calibration of the X-rays was performed using the *IPanalyzer* software (Seto *et al.*, 2010 ▸) by applying the double-cassette method with two XRD two-dimensional (2D) data of CeO_2_ (NIST SRM 674b) obtained beforehand using the imaging plate with varying detector distances.

**Figure 1:**
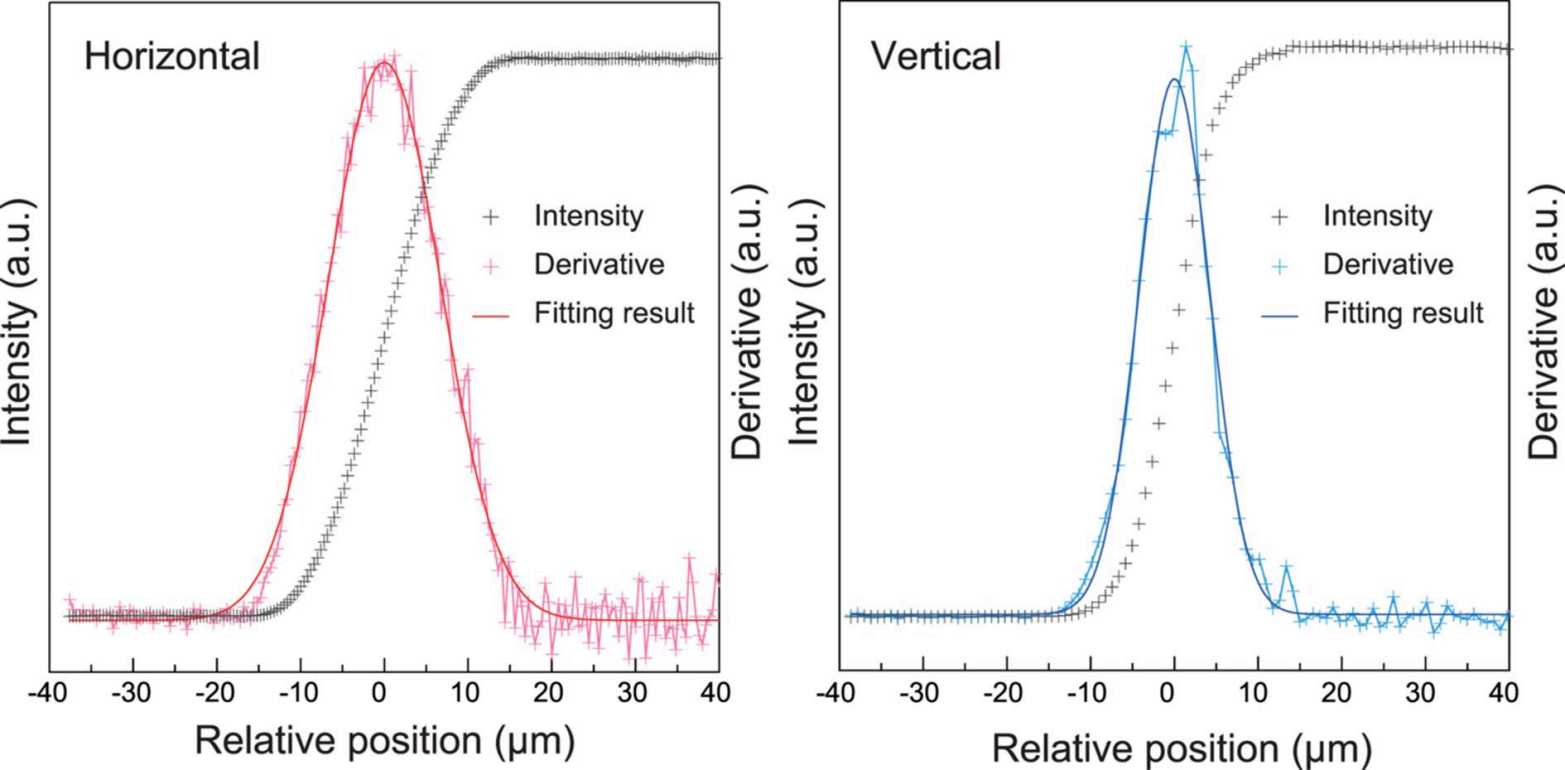
Results of measuring focused X-rays using the knife-edge method. Black crosses, pink/light blue crosses and solid red/blue lines indicate the measured raw values, their derivatives and the Gaussian fitting results of derivatives, respectively.

The sample in the DAC was extremely small, with a diameter of 100 µm and thickness of <10 µm. In particular, given that the spot size of the laser used for heating is approximately or less than 40 µm in diameter, it is generally necessary to use focused X-rays when collecting XRD data from high-pressure samples in DACs. At BL10XU/SPring-8, an X-ray compound refractive lens has traditionally been used as the X-ray focusing optical system (Ohishi *et al.*, 2008 ▸; Hirao *et al.*, 2020 ▸). Fig. 1 ▸ presents the results of the focused X-ray measurements using the knife-edge method. The X-ray beam size was 6.5 µm in the vertical direction and 9.5 µm in the horizontal direction (full width at half-maximum). In this case, the flux at the sample location was measured to be 1.1 × 10^12^ photons s^−1^.

**Figure 2:**
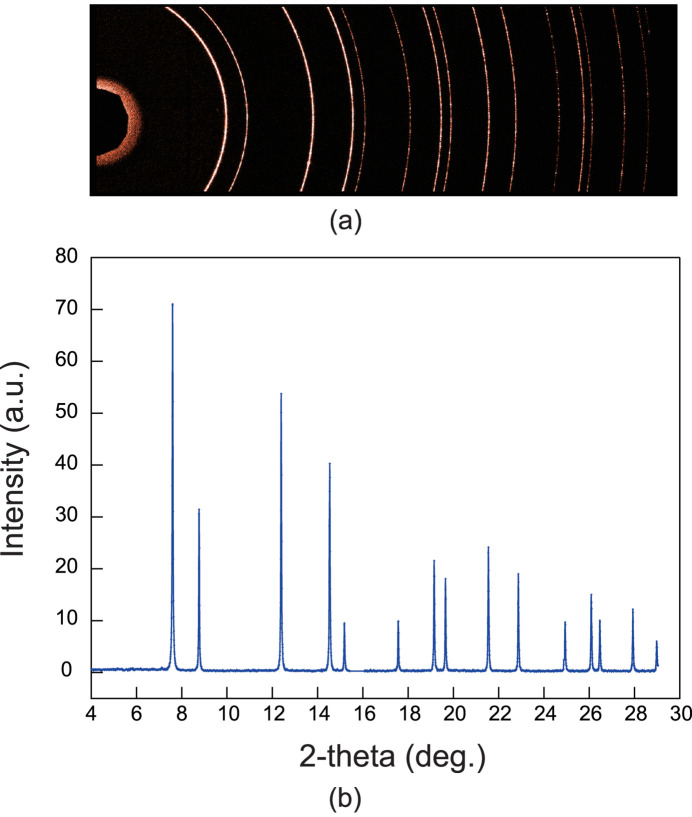
(*a*) Two-dimensional XRD data of CeO_2_ obtained using the LAMBDA 750k detector by applying focused X-rays at 30 keV; (*b*) results of integrating (*a*) into one dimension.

XRD measurements for CeO_2_ were performed using focused X-rays, and the sample dimensions were similar to those used in DACs. The two-dimensional XRD image was acquired with an exposure time of 1 ms using the LAMBDA 750k detector. The imaging mode of the detector was a single-threshold 12-bit mode, which enables continuous measurement with zero dead-time between acquisition frames. Fig. 2 ▸(*a*) shows the two-dimensional XRD data obtained using the detector and Fig. 2 ▸(*b*) shows the results of integrating the two-dimensional data into a one-dimensional powder diffraction pattern. A Python code created using the *pyFAI* library (Ashiotis *et al.*, 2015 ▸; Kieffer *et al.*, 2020 ▸) was used to integrate the two-dimensional XRD data and convert them into one dimension. These data indicated that, by using the high-brilliance synchrotron X-rays at SPring-8, data that are sufficient for analysis can be obtained within short exposure times of 1 ms, even when using the focusing optical system.

The synchronization of the LAMBDA 750k detector, temperature and pressure control system, and temperature measurement system using the spectroradiometric method was achieved by sending an external trigger signal to each device. Using multiple DG645 digital delay pulse generators (Stanford Research Systems), a trigger signal with an arbitrary delay and time width could be sent for each device. This allowed for the control of the start and end times of heating/rapid-cooling, compression/rapid-decompression and temperature measurement. A notable feature of the present system is that it allows easy integration of other measurement and external field control devices, such as those for electrical resistance measurement or power sources for resistance heating. In the subsequent sections, we present the development of the sample environment systems and their synchronization with the high-speed XRD measurement system combined with microfocus X-rays.

### 2.2. Upgrade of flat-top laser heating optics and construction of an experimental system for instantaneous laser heating

We visualized the structural changes in samples during instantaneous heating using a laser heating system with the high-speed XRD measurement system. In high-pressure and temperature experiments employing DACs, laser heating has been the mainstream method owing to the transparency of diamond. At BL10XU/SPring-8, we achieve and measure temperatures ranging from 1500 to 6000 K using a double-sided laser heating system.

The setup comprises two fiber lasers (SP-100C, SPI Lasers) that operate at a wavelength of 1064 nm and produce an output of up to 100 W. For the optical system employed for sample observation, we use infinity corrected optics. We employed lenses with a focal length of 80 mm and 1058 mm as the objective and imaging lens, respectively. These lenses have been specially designed to reduce aberration in the wavelength range 600–800 nm (Opt Sigma). The maximum chromatic aberration was 630 µm, which was controlled to ∼27% of the depth of the focus (2.31 mm). The maximum distortion value was 0.024%. Furthermore, in terms of wavefront aberration, the design exceeded a Strehl definition of 0.95 at each wavelength, which represents the maximum intensity ratio of point image distribution, thus forming an aberration-free lens optical system.

**Figure 3:**
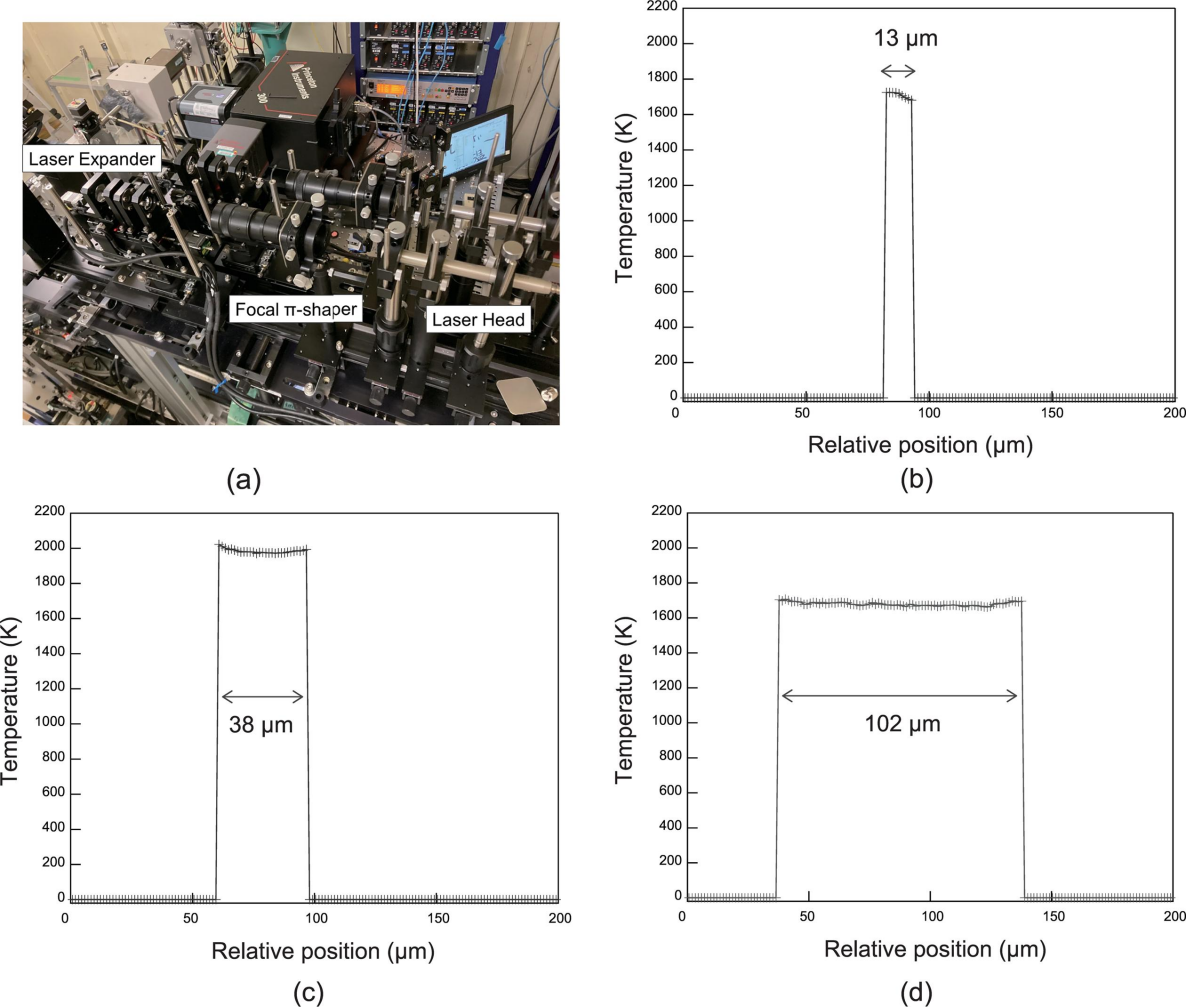
(*a*) Photograph of the flat-top laser heating system at BL10XU/SPring-8; (*b*)–(*d*) temperature profiles of test heating a platinum foil with various laser spot sizes.

The objective lens also functioned as a focusing lens for the lasers. In a high-temperature experiment using a laser heating system, the homogeneity of the sample composition and accuracy of the physical property measurement data are strongly dependent on the temperature gradient. In instantaneous heating experiments particularly, it is not possible to adjust the position of the laser during the heating process. Consequently, improving the uniformity of heating becomes crucial for the success of the experiment. Recently at BL10XU/SPring-8, we designed an optical system by combining a focal π-shaper (Focal-πShaper 9_1964, Adl­Optica) and laser expander to achieve flat-top laser heating [Fig. 3 ▸(*a*)]. The focal π-shaper is an optical system that realizes flat-top heating at the focus position when a focusing lens is used; it has been utilized in various laboratories and beamlines (Prakapenka *et al.*, 2008 ▸; Anzellini *et al.*, 2018 ▸). The motorized laser expander, which was designed for BL10XU/SPring-8 by OptoSigma, was installed immediately after the π-shaper. Figs. 3 ▸(*b*)–3(*d*) show the temperature measurement results of the test heating of a platinum foil. By combining these devices, it is possible to perform flat-top heating for up to 15–100 µm diameter at the sample position.

Temperature measurements during laser heating were performed by a spectroradiometric method using HRS-300-SS and ProEM-HS:512B eXcelon (Teledyne Princeton Instruments) as a spectrometer and detector, respectively. Using the ProEM detector, it is currently possible to perform high-speed imaging up to 67.4 frames s^−1^. The detector is integrated into a high-speed XRD measurement system and data collection is initiated by an external trigger signal.

The laser output was controlled by an analog output of 0–10 V using direct current (DC) regulated power supplies. When we first launched the high-speed XRD measurement system, we controlled the timing of laser emission by sending a trigger signal to the regulated power supplies (KX-100L, TAKASAGO Ltd). Subsequently, we connected a function generator to the laser oscillator and realized faster-response heating experiments by transmitting a pulse signal from the function generator (EDU33210A, Keysight Technologies). In Section 3, we present the experimental results obtained using the stabilized power supply and function generator as well as a comparison of the two approaches.

### 2.3. Development of the two-line gas-pressure control system for remote and rapid compression–decompression experiments

**Figure 4:**
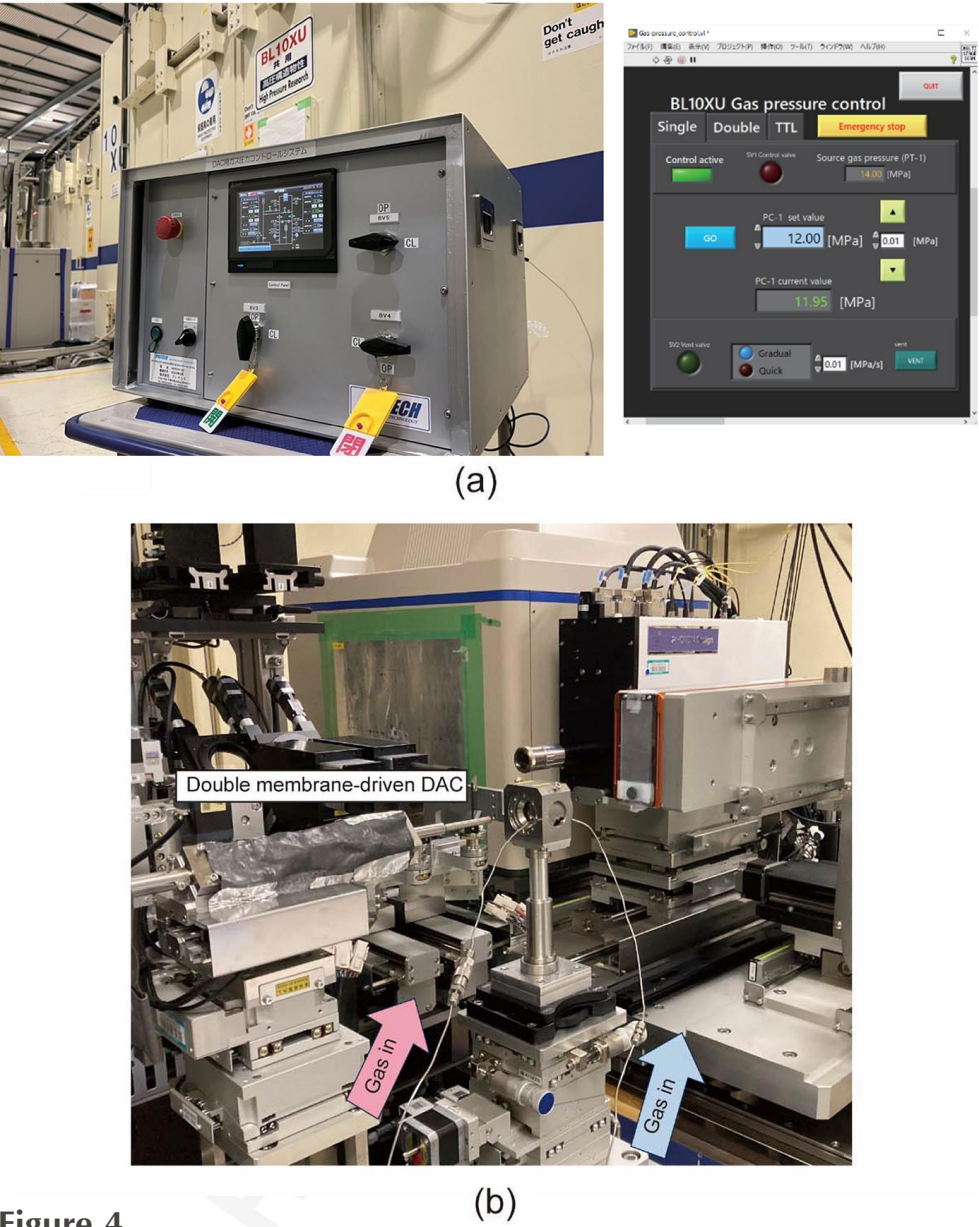
Photographs of (*a*) the two-line gas-pressure control system (left) and a screenshot of its control software based on LabVIEW (right); (*b*) a double-membrane-driven DAC mounted on the sample stage. Pink and blue arrows indicate that gas lines to supply gas pressure to the membrane for compression and decompression, respectively.

A new two-line gas-pressure control system was developed to enable remote pressure control and perform rapid compression–decompression experiments. Compression and decompression are unavoidable procedures in DAC experiments. They are used as the general method to manually increase or decrease pressure using screws and gearboxes located outside of the experimental hutches. Thus, each instance of pressurization or depressurization requires entry and exit from the hutch, a process that is complex and remarkably time-consuming. One approach for realizing remote pressure control is to use a membrane-driven DAC. A membrane-driven DAC is a type of DAC wherein a membrane (like a metal balloon) is attached to the piston side of the DAC. As gas pressure is applied to inflate the membrane, the piston is driven to pressurize the sample. At BL10XU/SPring-8, a single-line gas-pressure control system to increase the pressure was installed; however, due to the resistance between the DAC piston and cylinder, it was difficult to control pressure in during decompression using only gas-pressure reduction in the membrane. Therefore, it was difficult to precisely assess phase equilibrium during decompression. Furthermore, in low temperature experiments, pressure is often observed to increase owing to the thermal contraction of the DAC. As mentioned above, it is almost impossible to precisely decrease the pressure; therefore, users have to constantly monitor the sample pressure. Above all, conventional gas-pressure control systems are not equipped with a mechanism for rapidly changing the gas pressure, making rapid pressurization and depressurization experiments impossible. To resolve these experimental difficulties, a two-line gas-pressure control system was developed in FY2021 in collaboration with PRETECH Co. Ltd. Fig. 4 ▸(*a*) shows the newly introduced two-line gas-pressure control system and its control software based on LabVIEW and Fig. 4 ▸(*b*) shows a double membrane-driven DAC (SYNTEK Co. Ltd) mounted on a sample stage and connected to a gas line for gas-pressure supply. The gas pressure from the gas cylinder is decreased to the target pressure using the gas-pressure regulator and mass flow controller in the gas-pressure controller and supplied to the DAC at a specified flow rate.

Using this system, a maximum gas pressure of 18 MPa can be safely supplied. The pressure regulator and exhaust are electrically operated open or close valves, which can be remotely controlled by the control panel on the main unit and the user graphical user interface from the control computers. The system is equipped with two gas line systems for separately applying gas pressure. The new gas control system combined with a double-membrane-driven DAC, a DAC that enables depressurization control by pushing the cylinder side with a membrane, can achieve conventional compression experiments and precise decompression-control experiments. This new gas-pressure controller system can be easily synchronized with XRD measurement equipment via the main control system of the BL10XU/SPring-8. In Section 3.3, we provide the actual measurement results of the fast compression experiment with this new gas control system.

## 3. Submillisecond XRD measurements during changing temperature and pressure

### 3.1. Rapid laser heating and cooling experiments with laser power control via regulated power supplies

In this section, we present the results of an example of a laser-driven rapid heating and quenching experimental setup with the laser power being controlled by regulated power supplies, focusing on the instantaneous heating of synthetic fayalite (Fe_2_SiO_4_). Instantaneous heating is undoubtedly a valuable experimental technique primarily aimed at preventing reactions and contamination from the surrounding pressure medium and anvil. Additionally, it serves as a tool for research. Approximately 4.6 billion years ago, Earth was covered with a magma ocean, and the crystallization and segregation of this magma led to the formation of the present stratified Earth. The order of crystallization in the magma ocean has been discussed to understand the chemical and thermal evolution of Earth. Furthermore, there exists an anomalous structure at the base of the present Earth’s mantle, known as the ultralow velocity zone (ULVZ), where seismic wave velocities are slower than their surroundings. The possibility that ULVZ could represent the residue of silicate-oxide magma that came from the magma ocean has been a topic of longstanding discussion (Labrosse *et al.*, 2007 ▸). If ULVZ indeed represents a residue of the magma ocean that is still maintained in a liquid state today, this would imply that this surviving residue of the magma ocean continues to crystallize and interact with the surrounding mantle and outer core to date. Herein, we aim to visualize the crystallization process of the silicate melt, which is crucial for elucidating the evolution of Earth, using *in situ* time-resolved XRD measurements. Previous studies that attempted to clarify the liquidus phase of silicate melts adopted the method of *ex situ* chemical analysis of the recovered samples after high-temperature and pressure experiments (Nabiei *et al.*, 2021 ▸). To date, there have been no actual *in situ* observations under high-temperature and pressure conditions.

**Figure 5:**
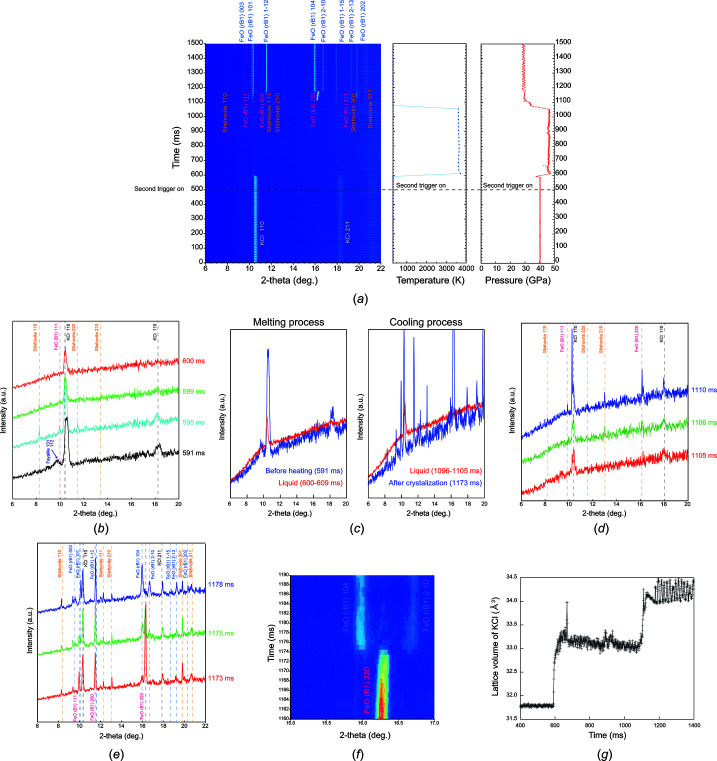
(*a*) XRD data collected continuously after every millisecond, temperature data and calculated pressure from the lattice volume of the KCl B2 phase with the *P*–*V*–*T* EoS (Tateno *et al.*, 2019 ▸); (*b*) process of melting; (*c*) comparison of the halo patterns of the liquid before heating (591 ms) and immediately after melting (10 data averaged from 600–609 ms), and the halo pattern of the liquid just before quenching (10 data averaged from 1096–1105 ms) with the XRD pattern during cooling (1173 ms), respectively; (*d*) process of crystallization of stishovite and the B1 phase of FeO from liquid Fe_2_SiO_4_; (*e*, *f*) phase transition from the B1 to rB1 phase of FeO during cooling; (*g*) time variation of lattice volume of the KCl B2 phase during heating and cooling.

Herein, we conducted instantaneous melting experiments on fayalite, as an Fe end component of the Mg_2_SiO_4_–Fe_2_SiO_4_ system, a mineral representative of those constituting the Earth’s mantle and meteorites. These experiments were conducted in six runs under pressures of 28.6–52.0 GPa (Table 1 ▸). The details of a typical run are as follows. The synthesized fayalite was used as the starting material. The sample was made into a pellet of approximately 50–80 µm diameter and 20 µm thickness. The sample pellet was loaded with dry potassium chloride (KCl) powder, which acted as a pressure medium and thermal insulator, through a sample hole of 100 µm diameter drilled into a rhenium gasket of 40–50 µm thickness. The sample was then compressed up to desired pressures using diamond anvils using a 300 µm-diameter culet. The first pulse triggered by DG645, a digital delay pulse generator, initiated XRD measurements on the LAMBDA 750k detector and temperature measurements on the ProEM-HS:512B eXcelon detector. Further, the second pulse was generated half a second after the first pulse. Synchronized with the second pulse, the lasers emitted light with an output of 30 W each from the upstream and downstream. The laser heating spot size of each was over 30 µm diameter. The sample was heated to a maximum temperature of 3270–3700 K in each run (Table 1 ▸), causing the sample to melt. The laser output was maintained for 0.5–1 s after emission; then, the laser was turned off. Fig. 5 ▸(*a*) shows the XRD data continuously collected at every millisecond, temperature data and calculated pressures. All XRD data were fitted using the Le Bail method, and the lattice parameters were calculated. Pressure was calculated from the lattice volume of the KCl B2 phase (space group: 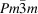 ) using the XRD peak from the Bragg reflection 110 with the *P*–*V*–*T* equation of state (EoS) (Tateno *et al.*, 2019 ▸). The temperature measurement results were interpolated between each data point by linear interpolation. Fig. 5 ▸(*b*) shows the XRD patterns with increasing temperature. The XRD peak intensity of fayalite decreases and the background of the data increases with increasing temperature. Then, 600 ms after the first pulse, a halo pattern originating from the liquid sample is observed, which indicates the complete melting of fayalite at 44.3 GPa and 3400 K. Fig. 5 ▸(*c*) compares the halo patterns of the liquid before heating (590 ms) and immediately after melting (10 data averaged from 600 to 609 ms), and the halo pattern of the liquid just before quenching (10 data averaged from 1096 to 1105 ms) with the XRD pattern during cooling (1173 ms), respectively. Experimental results also showed that the sample was still completely molten at 1105 ms after the experiment started. Stishovite (space group: *P*4_2_/*mnm*) was crystallized 1106 ms later. The FeO B1 phase (space group: 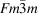 ) appeared 4 ms later at 1110 ms [Fig. 5 ▸(*c*)]. These results indicate that the liquidus phase of Fe_2_SiO_4_ at 44.3 GPa is stishovite. Further, we succeeded in observing the phase transition from the FeO B1 phase to the rB1 phase (space group: *R*3*m*) during cooling, which occurred 1173 ms after the start of the experiment [Figs. 5 ▸(*d*) and 5(*e*)]. Under all pressure conditions, stishovite crystallized first from the molten sample after laser quenching, followed by FeO crystallization 3–5 ms later. Therefore, we concluded that the liquidus phase of Fe_2_SiO_4_ was stishovite under all experimental pressure conditions. Our result is consistent with that of a previous study using *ex situ* chemical analysis (Kato *et al.*, 2016 ▸). We have thus demonstrated a new approach for *in situ* time-resolved XRD measurement under high pressure and temperature using laser-heated DACs. The developed system has been made available since 2021 and is currently yielding intriguing results (Ohta *et al.*, 2023 ▸). However, additional refinement was required regarding the fast-heating aspect within a few milliseconds, which is discussed below.

### 3.2. Millisecond-pulse laser heating with laser power control via a function generator

As discussed in Section 3.1, we developed a time-resolved XRD measurement system by incorporating regulated power supplies as a laser output control function. The relationship between the lattice volume of KCl calculated from the obtained XRD data and time course is shown in Fig. 5 ▸(*f*). The XRD data of Fe_2_SiO_4_ and the variation of the lattice volume of KCl (Fig. 5 ▸) indicate a time lag of 93 ms from the second trigger signal to the firing of the laser, and heating was initiated 593 ms after the occurrence of the first trigger signal. The sample was completely molten 598 ms after the first trigger. Following a heating time of 508 ms, the sample underwent rapid cooling 1105 ms after the first trigger signal, with a time lag of 105 ms from the falling edge of the second trigger signal. The duration from the moment the stabilized power supply initiated the reduction of voltage until the sample has completely cooled was estimated to be ∼40 ms, and the pulse rising and falling times of the digital delayed pulse generator were set to 1 ns. The time lag and rise and fall of temperature with a rising and falling time of ∼40 ms are the same for all experiments using this device. Furthermore, it is impossible to perform heating for <100 ms using this system.

**Figure 6:**
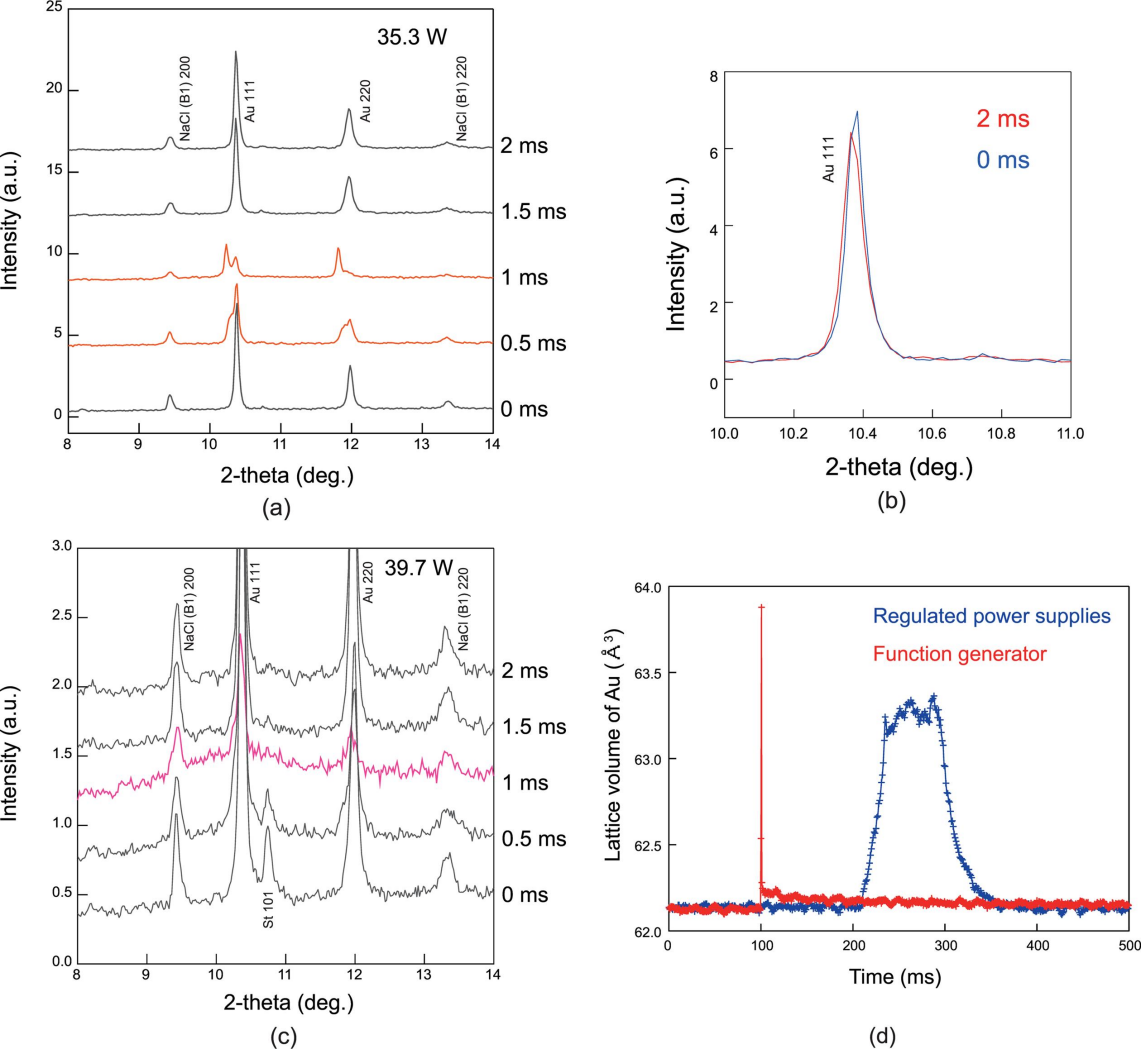
(*a*, *c*) Results of continuous XRD measurements at 0.5 ms under 1 ms pulse heating. The laser output is combined with upstream and downstream power of 35.3 and 39.7 W, respectively. Orange lines represent XRD data during pulse heating with a laser power of 35.3 W. The broad diffraction peak derived from the halo pattern of the liquid sample can be confirmed using XRD data at high temperatures (pink line); (*b*, *d*) time variation of lattice volume of Au: a comparison of the experimental results from the rapid heating system with a 100 ms time width using the regulated power supplies (blue symbols) and that of 1 ms pulse heating using a function generator (red symbols).

Further, to control the laser heating duration more precisely and evolve the system to one that allows short pulse heating and a flexible temperature rise, we installed a function generator for laser output control instead of a stabilized power supply. To test the pulse heating performance of the function generator, experiments were conducted as described below. NaCl, SiO_2_ and Au pellets were loaded as samples in a gasket with a hole diameter of 100 µm, and a pressure of 20.3 GPa was applied at room temperature using diamond anvils with a culet size of 300 µm. The prepared samples were shot-heated multiple times at different heating points. Figs. 6 ▸(*a*) and 6(*b*) show the results of continuous XRD measurements at 0.5 ms under 1 ms pulse heating. The laser output was combined with upstream and downstream power of 35.3 and 39.7 W, respectively. The laser heating spot size had a diameter of >30 µm, which is sufficiently smaller than the size of a focused X-ray beam (see Section 2.1). In the heating experiment depicted in Fig. 6 ▸(*a*), the response of Au to 1 ms pulses of laser heating can be observed from the changes in XRD peaks derived from the 111 and 220 Bragg reflections. Furthermore, these XRD peaks were split by heating. After heating, the 111 Bragg reflection peak positions of Au returned to their original scattering angle positions. A magnified view comparing the 111 peak positions of Au immediately after the start of triggering and after 2 ms is shown in Fig. 6 ▸(*b*). The scattering angle positions were 10.378 (1)° and 10.371 (1)°, respectively. This implies that the heating process was completed before heat was uniformly diffused into the sample by laser heating. In addition, a halo pattern observed in the experiment, where heating was performed at higher laser output powers, indicated that Au could be melted with 1 ms pulse heating. In this run, the sample completely cooled and solidified ∼2 ms after heating started [Fig. 6 ▸(*b*)]. As shown in Fig. 6 ▸(*c*), through the time variation data of the lattice volume of Au, we compared the experimental results from the rapid heating system with 100 ms heating time using the regulated power supplies introduced in Section 3.1 and the new system that enabled 1 ms pulse heating using the function generator. Using regulated power supply, a delay of ∼100 ms was generated before heating started, and needed >40 ms to increase and decrease the temperature. These issues were solved using the function generator.

Thus, instantaneous heating is beneficial for experiments aimed at dynamic heating and to prevent reactions and contamination from surrounding anvils and pressure media. Prior to the development of the present systems, numerous studies were conducted at BL10XU/SPring-8 involving manually controlled instantaneous heating. The laser was forced to stop in ∼1 s or less without strict time control. However, the only means of estimating what was occurring during heating was through ex-heating XRD measurements and chemical analysis of the recovered samples. Here, we conducted continuous XRD measurements with a submillisecond exposure time, which enabled us to clarify for the first time the relationship between the passage of time and events within traditional instantaneous heating without time strict control, *i.e.* experiments in which the experimenter measures time and turns off the laser with a manual button. Furthermore, the refined control of the heating time certainly expanded the possibilities for laser heating experiments under high pressure using DACs at BL10XU/SPring-8.

### 3.3. Fast compression and decompression experiments using a membrane/double-membrane-driven DAC and gas-pressure control system

As an example of the application of the submillisecond XRD measurement system developed in this study, we introduced the results of the rapid compression–decompression experiments. The experiments were performed using the developed gas-pressure controller introduced in Section 2.3. Au, commonly used as a pressure marker, and NaCl, which also serves as a pressure marker and pressure medium, were selected as samples in the rapid compression–decompression experiments. A hole of 100 µm was drilled in Re gaskets that were pre-indented to a thickness of ∼40 µm, and the loaded Au pellet was sandwiched between the NaCl pellets. The samples were compressed using 300 µm culet diamond anvils. The pressure was calculated from the EoS (Tsuchiya, 2003 ▸) using the lattice volume obtained from the diffraction peak derived from the 111 Bragg reflection of Au.

**Figure 7:**
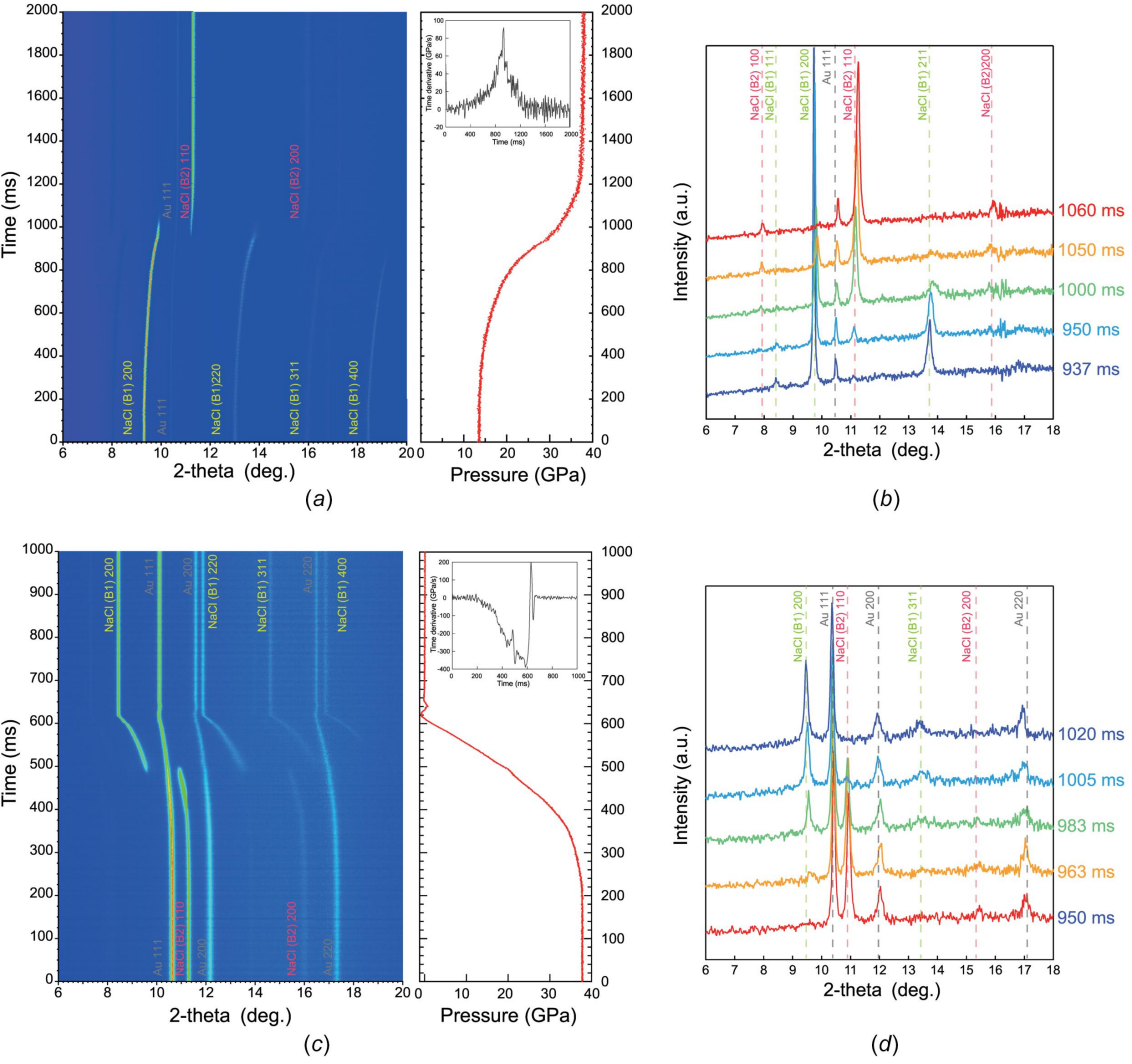
(*a*, *c*) A series of *in situ* XRD data under fast compression–decompression experiments, and the relationship between pressure and time derived from lattice volume changes of Au and its time derivative; (*b*, *d*) 1D XRD patterns under fast compression–decompression experiments.

Before performing rapid compression, a gas pressure of 0.86 MPa was supplied to attach the membrane to the piston part, and the initial pressure of the sample was 13.5 GPa. In the rapid compression experiment, the input gas-pressure value was set to 8 MPa on the gas-pressure controller. The Time to Live (TTL) signal that was used to open the valve of the gas-pressure controller was simultaneously input with the first signal to initiate the XRD measurement without delay. Fig. 7 ▸(*a*) shows the time variation of a series of *in situ* XRD data, and the obtained results and the relationship between pressure and time are determined based on lattice volume changes of Au. It can be observed that, 937 ms after the valve was opened, the sample pressure reached 28.8 GPa, which subsequently triggered the high-pressure phase transition from the B1 to B2 phase of NaCl [Fig. 7 ▸(*b*)]. Furthermore, the intensity of the diffraction peaks derived from the B1 phase gradually decreased, while those from the B2 phase increased. Further, we observed a series of events *in situ* in which the high-pressure phase transition of NaCl was completed at 34.2 GPa, 1060 ms after the initiation. The change in pressure with time shown in Fig. 7 ▸(*a*) can be interpreted as a gradual application of pressure to the sample, commencing 230 ms after the release of the valve. The gas-pressure controller was installed outside the experimental hutch, and it is necessary to traverse a 15 m stainless tube with a 1/16-inch diameter to supply gas to the membrane attached to the DAC. This caused a delay until the sample was pressurized. The pressure slowly increased from 13.5 to 19 GPa over 520 ms, after which an enhancement in pressurization efficiency was observed. The maximum compression speed of 76 GPa s^−1^ was achieved at around 916 ms [Fig. 7 ▸(*a*)].

To conduct the rapid decompression experiment, we introduced gas pressure into the membrane for compression at 6 MPa, which increased the sample pressure to 37.7 GPa. A gas pressure of 5 MPa was applied to the gas line leading to the membrane for decompression, while keeping the valve between the membrane and the gas line closed. The TTL signal was input to open the leak valve of the membrane for pressurization simultaneously with the XRD trigger. A 100 ms delay from the XRD trigger was given to open the valve between the membrane for decompression and the gas pressure control in order to apply gas pressure into the membrane for decompression. Figs. 7 ▸(*c*) and 7(*d*) show a series of *in situ* XRD measurements of rapid decompression experiments. In the decompression experiment, each XRD data set was collected with an exposure time of 0.5 ms. XRD peaks from the B1 phase of NaCl began to be observed at 20.2 GPa, 491.5 ms after the initial trigger; after 510 ms, XRD peaks from the B2 phase of NaCl completely disappeared at 17.2 GPa. The maximum decompression speed of 355 GPa s^−1^ was reached around 573 ms after the first trigger [Fig. 7 ▸(*c*)].

Herein, we aimed to generate relatively moderate pressure using a diamond anvil with a 300 µm culet to precisely observe the phase transition of NaCl. We anticipate that, by using an anvil with a smaller culet in the future, our system can achieve a considerably high compression–decompression speed (Jenei *et al.*, 2019 ▸).

## 4. Conclusion

In an endeavor to simulate and study the reaction processes under high pressure and temperature changes witnessed in extreme dynamic conditions, such as those observed in the case of meteorite impacts, we developed a technique for submillisecond *in situ* XRD measurements with changing pressure and temperature using DACs at BL10XU/SPring-8. In particular, system development involved three key aspects: (1) the development of a high-speed XRD measurement system combined with microfocus X-rays, (2) upgrading of flat-top laser heating optics and construction of an experimental system for instantaneous laser heating, and (3) development of a two-line gas-pressure control system for remote and rapid compression–decompression experiments. The high-speed XRD measurement system is a system built around the recently installed LAMBDA 750k CdTe detector at BL10XU/SPring-8. It is a hybrid pixel array detector employing CdTe sensors, providing a high angular resolution owing to its small pixel size of 55 µm × 55 µm and enabling fast continuous measurements up to 2 kHz in a noise-free mode. High-speed XRD measurements of tiny samples in DACs were successfully performed by synchronizing the LAMBDA 750k CdTe detector with multiple devices to dynamically control the sample environment, such as temperature and pressure, and combining it with the focused X-ray generation technique developed at BL10XU/SPring-8. For the optimization of laser heating for its incorporation in the high-speed XRD measurement system, an electron multiplying CCD with high sensitivity and speed was introduced for radiometric temperature measurement to realize fast temperature measurements over 50 Hz. Furthermore, the inhomogeneity of heating areas poses a serious problem in laser heating experiments. In particular, the influence of this inhomogeneity is remarkable in high-speed XRD measurements using a microfocus X-rays of size <10 µm, similar to the finding of this study. Therefore, to overcome this problem, a novel laser beam homogenization system was developed by employing focal π-shapers. Consequently, the temperature inhomogeneity within the X-ray measurement spot, which was >20% when using the conventional system, was reduced to <5%. Herein, we also aimed to achieve rapid pressure changes using membrane-driven DACs as well as developed and introduced a gas-pressure controller capable of rapid compression and decompression, equipped with a mechanism for opening and closing each solenoid valve using a TTL signal, thereby enabling remote control through remote operation functions from the control system. This allowed the realization of high-speed compression–decompression experiments with speeds of >76 GPa s^−1^ and >355 GPa s^−1^, respectively. A primary advantage of this system lies in its versatility. In addition to changing temperature and pressure, it can seamlessly integrate various composite measurements, such as those involving physical properties, as exemplified by electrical resistance measurements (Ohta *et al.*, 2023 ▸). Furthermore, it can be easily applied to experiments assessing the sample behavior under complex environmental changes. Our future objective entails the development of a measurement system that targets smaller samples, anticipating the utilization of high-brilliance X-rays derived from low-emittance electron beams following the SPring-8-II upgrade. In addition, we aim to broaden the capabilities of our system to address a wide range of requirements, such as combining sample environment applications that go beyond just temperature and pressure and synchronizing composite measurements with physical property measurements, such as thermal conductivity with pulsed light heating thermo­reflectance technique (Ohta *et al.*, 2012 ▸; Imada *et al.*, 2014 ▸).

## Figures and Tables

**Table 1:** Pressure and temperature conditions, unit-cell volumes of KCl B2 at room temperature and at high temperature just before temperature quenching for rapid laser heating and cooling experiments of Fe_2_SiO_4_

Run number	Initial pressure (GPa)	Initial lattice volume of KCl (Å^3^)	*In situ* temperature (K)†	Frame rate of temperature measurements	*In situ* pressure (GPa)‡	*In situ* lattice volume of KCl (Å^3^)
1	19.7	37.05	3260 (80)	54.6	28.6 (0.2)	37.88
2	31.5	33.61	No temperature measurement data	No temperature measurement data	No temperature measurement data	33.83
3	37.4	32.32	3410 (150)	67.4	41.9 (0.6)	33.96
4	37.8	32.24	3500 (80)	40.8	44.3 (0.2)	33.48
5	42.3	31.97	3680 (120)	25.8	51.2 (0.4)	32.18
6	39.2	31.42	3640 (120)	25.8	52.2 (0.4)	31.97

† The temperature uncertainty is provided by the average temperature variation over a heating area of 26 µm.

‡ Pressure uncertainty is calculated based on temperature variations.
